# Construction of a TTN Mutation-Based Prognostic Model for Evaluating Immune Microenvironment, Cancer Stemness, and Outcomes of Colorectal Cancer Patients

**DOI:** 10.1155/2023/6079957

**Published:** 2023-02-21

**Authors:** Lei Zhao, Weiwei Fan, Kunpeng Luo, Siqi Xie, Rui Wang, Jingming Guan, Zhendong Chen, Shizhu Jin

**Affiliations:** ^1^Department of Gastroenterology and Hepatology, The Second Affiliated Hospital of Harbin Medical University, Harbin, China; ^2^Department of Infectious Medicine, Heilongjiang Provincial Hospital, Harbin, China

## Abstract

**Background:**

Colorectal cancer (CRC) is one of the commonest cancers worldwide. As conventional biomarkers cannot clearly define the heterogeneity of CRC, it is essential to establish novel prognostic models.

**Methods:**

For the training set, data pertaining to mutations, gene expression profiles, and clinical parameters were obtained from the Cancer Genome Atlas. Consensus clustering analysis was used to identify the CRC immune subtypes. CIBERSORT was used to analyze the immune heterogeneity across different CRC subgroups. Least absolute shrinkage and selection operator regression was used to identify the genes for constructing the immune feature-based prognostic model and to determine their coefficients.

**Result:**

A gene prognostic model was then constructed to predict patient outcomes; the model was then externally validated using data from the Gene Expression Omnibus. As a high-frequency somatic mutation, the titin (TTN) mutation has been identified as a risk factor for CRC. Our results demonstrated that TTN mutations have the potential to modulate the tumor microenvironment, converting it into the immunosuppressive type. In this study, we identified the immune subtypes of CRC. Based on the identified subtypes, 25 genes were selected for prognostic model construction; a prediction model was also constructed, and its prediction accuracy was tested using the validation dataset. The potential of the model in predicting immunotherapy responsiveness was then explored.

**Conclusion:**

TTN-mutant and TTN-wild-type CRC demonstrated different microenvironment features and prognosis. Our model provides a robust immune-related gene prognostic tool and a series of gene signatures for evaluating the immune features, cancer stemness, and prognosis of CRC.

## 1. Introduction

Colorectal cancer (CRC) is one of the most common cancers worldwide and accounts for 9.4% of morbidity from all cancers. More than 1.9 million new CRC cases and 935,000 related deaths were estimated to have occurred in 2020 [1]. The incidence of CRC is related to the level of development of countries, with incidence rates in transitioning countries reported to be only one-quarter of that in transitioned countries [1]. Therefore, CRC may be considered a marker of socioeconomic development. The incidence rates of CRC are steadily increasing in many countries [2, 3]. Surgery, chemotherapy, and radiotherapy are standard conventional therapies for CRC; these therapies are combined to increase the efficacy of treatment. Nevertheless, CRC still could not be completely cured. In this context, relapse occurs in more than half of these patients even after neoadjuvant therapy [4]. In addition, these treatments are associated with certain side effects due to the lack of specificity, unfavorable combinations, and toxicities [5, 6]. Thus, it is essential to find strategies to improve the treatment and prognosis of CRC.

CRC is characterized by mutation accumulation and immune response dysregulation [7]. Somatic mutations are widely found in CRC; mutations of certain genes including KRAS, p53, SMAD4, and BRAF are associated with progression and metastasis of CRC [8]. Some mutations are found to be related to outcomes in patients with CRC; for example, AXIN2 variations are associated with poor prognosis [9]. KRAS mutations are known to contribute to the initiation of CRC and are used as a predictive biomarker for survival in these patients [10].

The immune microenvironment plays an important role in the progression of CRC [11–13]. As immunotherapy constitutes standard treatment for the disease [14], it is essential to improve understanding on the immune status of these tumors to improve treatment. Numerous studies have investigated the relation between the immune system and CRC; infiltration by immune cells is a prognostic biomarker in this disease [15]. Ge et al. demonstrated the immune infiltration and related gene expression profiles in CRC [16]; their findings may aid the selection of therapeutic targets and provide individualized therapeutic strategies. Notably, genomic alterations, such as those in BCL9L, RBM10, CTCF, and KLF5, correlate with immune cell infiltration in CRC [17].

In the present study, we obtained somatic mutation data of CRC patients from The Cancer Genome Atlas (TCGA) database and analyzed immune microenvironment changes mediated by somatic mutations. We also constructed an immune-feature based prognostic model for predicting immune infiltration features, immunotherapy responsiveness, cancer stemness, and the outcomes of patients with CRC.

## 2. Materials and Methods

### 2.1. Data Acquisition and Processing

For the training set, data pertaining to somatic mutations, gene expression profiles, and clinical parameters were acquired from the colon adenocarcinoma (COAD) and rectum adenocarcinoma (READ) TCGA cohorts. TCGAbiolinks R package [18] was employed to obtain gene expression and clinical data. Somatic mutation data were downloaded and visualized using the maftools R package [19]. Independent cohorts were employed for externally validating the robustness of the prognostic model. The raw count data were normalized by the transcripts per million (TPM) method and then transformed to log2 (TPM + 1). Data pertaining to the gene expression profile and clinical parameters of the validation set were acquired from the Gene Expression Omnibus (GEO) dataset (https://www.ncbi.nlm.nih.gov/geo/). For the immunotherapy cohort, data was acquired from the clinic trail of atezolizumab (anti-PD-L1 agent) via R package “IMvigor210CoreBiologies.”

### 2.2. Acquisition of Differentially Expressed Genes and Gene Enrichment Analysis

The Limma R package was employed to obtain the differentially expressed genes (DEGs) under the threshold *P* value of <0.05 and fold change (FC) > 1.3 or <-1.3. Functional enrichment was performed to further analyze the underlying biological function of the DEGs. The Gene Ontology (GO) [20] dataset including molecular function, biological pathways, and cellular components and the Kyoto Encyclopedia of Genes and Genomes (KEGG) [21] dataset were enrolled for functional enrichment analysis. The clusterProfiler [22] package in R was employed to analyze the GO function of the DEGs and enrich the KEGG pathway.

### 2.3. Comparison of Tumor Microenvironment among Clusters

The CIBERSORT R package [23] was applied to calculate the infiltration proportion of the 22 immune cell subtypes. Normalized TCGA-COAD and TCGA-READ expression data were included for immune infiltration proportion analysis. The relative expression of 22 tumor microenvironment infiltrating cells was calculated in each sample. Cancer stemness was evaluated based on the one-class logistic regression according to [24] research (https://bioinformaticsfmrp.github.io/PanCanStem_Web/).

### 2.4. Identification of Immune Subtypes

Univariate Cox regression was first performed to compare the impact of the DEGs on patient outcomes between the TTN-mutant and wild-type groups. The three immune subtypes were then identified by consensus clustering analysis based on the selected genes. Consensus clustering analysis was performed by the ConsensusClusterPlus R package [25].

### 2.5. Construction of the Immune Feature-Based Prognostic Model (Immune Suppressive Score)

DEGs between the immune suppressive and immune active CRC subtypes were selected for prognostic model construction. Univariate Cox regression was employed to analyze the correlation between overall survival (OS) and gene expression levels. Genes with *P* values of <0.01 were selected for subsequent prognostic model construction. Features were selected and assessed for their contribution as independent prognostic factors for patient survival using least absolute shrinkage and selection operator (LASSO) regression analysis, which was performed by the glmnet R package. The prognostic model including 25 genes was constructed by multiplying the regression coefficients (*β*) derived from the LASSO regression model with their messenger ribonucleic acid (mRNA) expression levels. The ISS based on the model was calculated as follows:

(1) $$\begin{array}{c} \text{ISS}=\sum \text{G}\text{ene coefficient}*\text{Gene expression level}. \end{array}$$

The immune suppressive score (ISS) of each sample was calculated based on the prognostic model. Samples were divided into the high-ISS group and low-ISS group based on the optimal cut-off value which is determined by the R package “survival.” The receiver operating characteristic (ROC) curves of the high- and low-ISS groups were analyzed and visualized using the survivalROC R package.

### 2.6. Statistical Analysis

Statistical analysis was performed using R 3.6.3 (https://www.r-project.org/). Continuous variables were analyzed using the Wilcoxon and Kruskal-Wallis tests for paired samples and three groups, respectively. Patient outcomes in the different clusters were compared using the log-rank test. *P* < 0.05 was considered statistically significant, unless specified otherwise.

## 3. Results

### 3.1. TTN Mutations Were the High-Frequency Somatic Mutation Type That Correlated to Patient Prognosis

**Figure 1:**
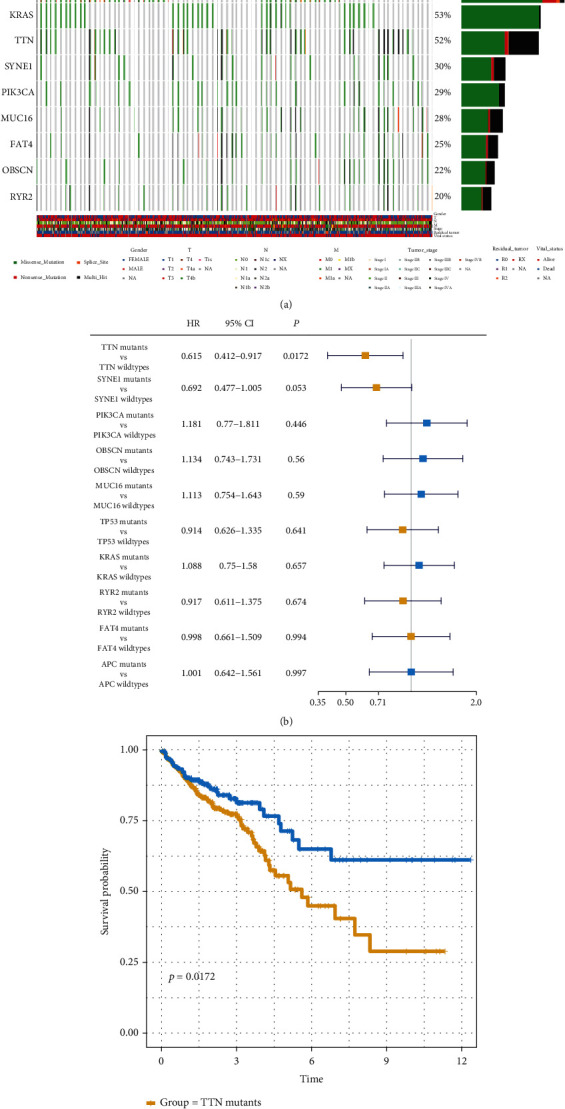
Correlation between TTN mutations and patient prognosis: (a) somatic mutation landscape of CRC patients; (b) correlation between patient prognosis and somatic mutations; (c) TTN mutations correlated with poor prognosis.

As shown in Figure 1(a), the 10 most common somatic mutations in CRC are found in APC, TP53, KRAS, TTN, SYNE1, PIK3CA, MUC16, FAT4, OBSCN, and RYR2.

Here, we analyzed the impact on the patient's prognosis of the 10 somatic mutation types (Figure 1(b)). Among the 10 mutations, only TTN mutations demonstrated significant impact on patient outcomes (log-rank *P* = 0.0172, hazard ratio (HR) = 0.615, 95% confidence intervals (CI) 0.412-0.917). On comparing patient prognosis between TTN-mutant and wild-type groups, we found the prognosis of the TTN-mutant group to be significantly poorer (Figure 1(c), log-rank *P* = 0.0172). This finding indicated that TTN mutations may represent a potential therapeutic target and index for prognosis in patients with CRC.

### 3.2. Analysis of TTN Mutation-Mediated Immune Characteristics

**Figure 2:**
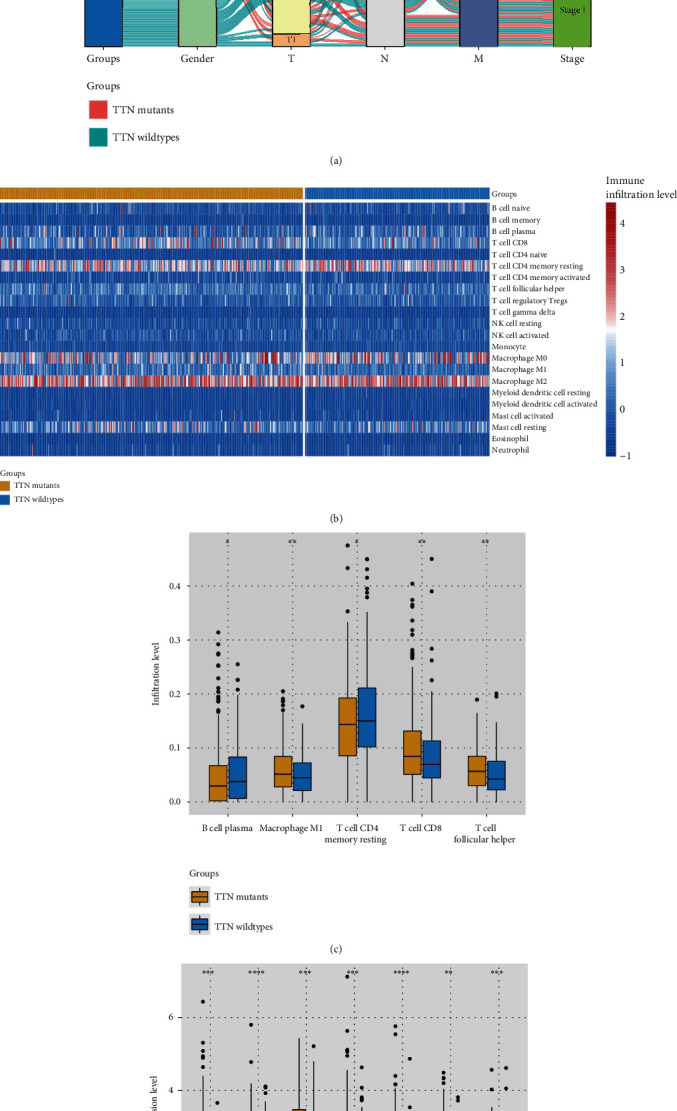
Influence of TTN mutations on the CRC immune microenvironment. (a) Sankey diagram representing the distribution of clinicopathological features in TTN-mutant and wild-type tumors; (b) immune infiltration status of TTN-mutant and wild-type tumors; (c) comparison of immune infiltration levels between TTN-mutant and wild-type tumors; (d) comparison of immune checkpoint expression levels in TTN-mutant and wild-type tumors.

Based on our findings, TTN-mutant CRCs were more likely to be advanced (Figure 2(a)). On further characterization of the immune microenvironment of TTN-mutant and wild-type cancers (Figure 2(b)), TTN-mutant tumors showed higher levels of CD8+ T cell infiltration and immune checkpoint expression, which inferred the immune microenvironment differences of TTN mutants and TTN wild types (Figures 2(c) and 2(d)). This implied that TTN mutation-mediated immune suppression of the microenvironment was caused by high levels of immune checkpoint activation. In this context, high levels of CD8+ T cell infiltration indicated potentially greater responsiveness to immunotherapy. Identification of the immune suppressive CRC subtype could therefore facilitate the selection of appropriate therapies for CRC in the clinic.

### 3.3. Identification of CRC Immune Subtypes

**Figure 3:**
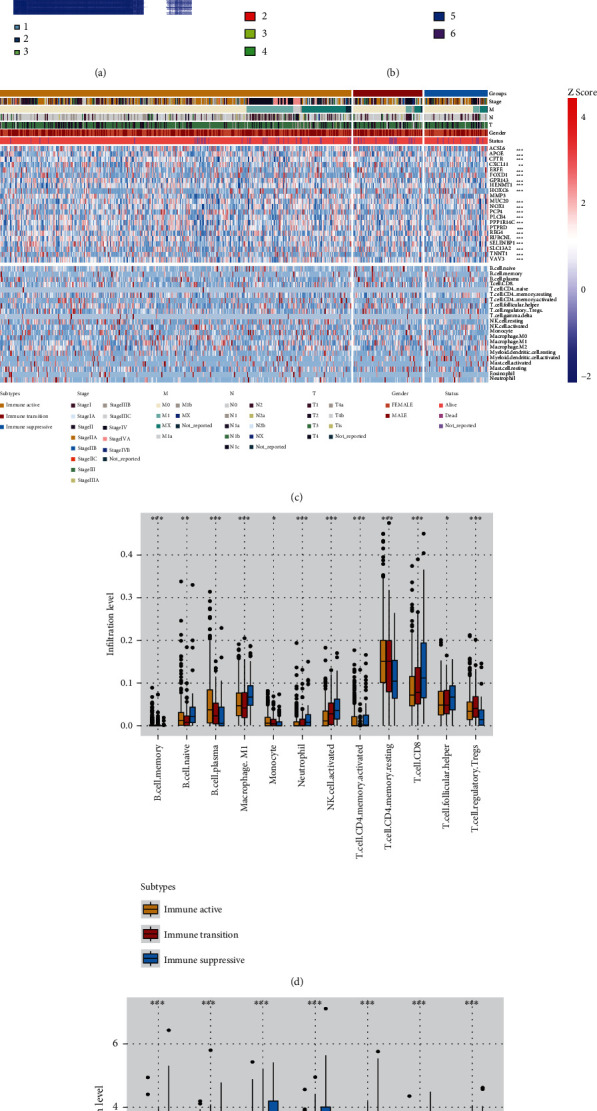
Identification of the three immune subtypes of CRC: (a, b) identification of CRC immune subtypes based on consensus clustering analysis; (c) immune landscape of the three CRC immune subtypes; (d) comparison of immune infiltration levels among the three immune subtypes; (e) comparison of immune checkpoint expression levels among the three immune subtypes.

As TTN mutants demonstrated a potentially immunotherapy-sensitive immune suppressive microenvironment, we further analyzed the biological heterogeneity between TTN-mutant and wild-type cases. First, we acquired the DEGs between the TTN-mutant and wild-type cases and performed enrichment analysis to analyze the biological functions mediated by the DEGs (Figure S1); DEGs related to patient prognosis were then identified. Overall, 22 genes were selected for subtype identification. Based on the findings, we performed consensus clustering analysis to identify the CRC immune subtypes (Figures 3(a) and 3(b)). This led to the identification of the immune active, immune transition, and immune suppressive CRC subtypes (Figure 3(c)). On comparing the immune infiltration level of the three CRC immune subtypes, we found that the immune suppressive subtype had the highest CD8+ T cell infiltration level among the three subtypes; this indicated the potential responsiveness of the immune suppressive CRC subtype to immunotherapy (Figure 3(d)). Our results also indicated that the immune suppressive subtype had the highest level of expression of the seven immune checkpoints (Figure 3(e)). This implied that the immune suppression in this subtype was mediated by high activity of the immune checkpoints and that the CD8+ T cells of the microenvironment may have an exhausted phenotype.

### 3.4. Construction and Validation of the Immune Feature-Based Prognostic Model

**Figure 4:**
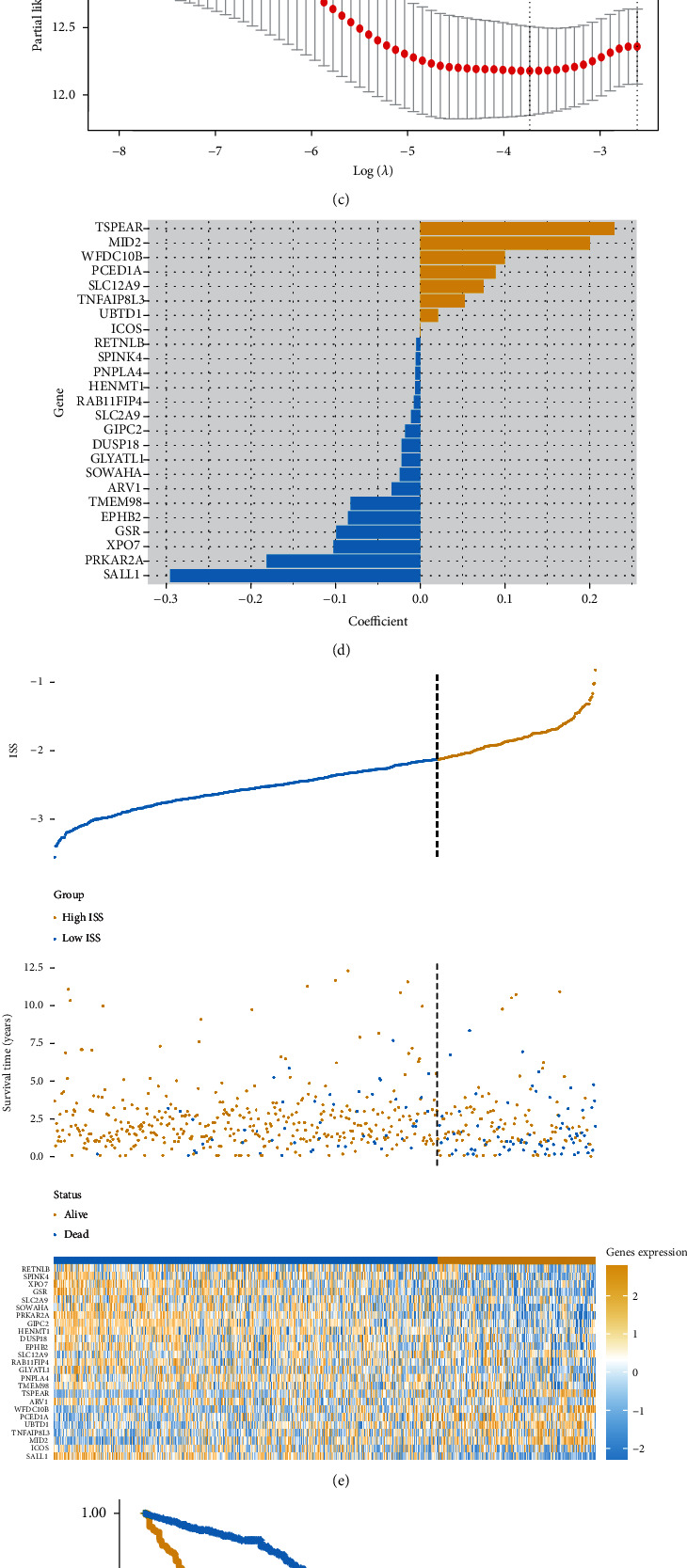
Construction of the immune feature-based prognostic model: (a) volcano plot representing the DEGs between the immune suppressive and immune active CRC subtypes; (b, c) LASSO regression was employed to construct the model; (d) coefficients of the selected genes; (e) distribution of the high- and low-ISS groups in the training cohort; (f) comparison of patient prognosis between the high- and low-ISS groups in the training cohort; (g) ROC curve for ISS in the training cohort.

We initially acquired DEGs between the immune suppressive and immune active CRC subtypes (Figure 4(a)). To further analyze the biological heterogeneity between the two subtypes, we employed enrichment analysis to explore the biological characteristics mediated by DEGs. The results of the enrichment analysis are shown in Figure S2.

To support clinical application, we constructed an immune feature-based gene prognostic model (providing an ISS) to predict patient outcomes using a machine learning-based method. Based on the LASSO regression results, 25 genes were selected to construct the gene prognostic model (Figures 4(b) and 4(c)); the regression coefficients of the 25 genes were then calculated (Figure 4(d)). To validate the efficiency of the ISS, we divided patients into high- and low-ISS groups (Figure 4(e)). Kaplan-Meier survival analysis demonstrated that patients with high ISS had significantly poorer outcome (Figure 4(f), *P* < 0.0001). The result further validated the prediction efficiency of the ISS. ROC analysis indicated that the model had best prediction efficiency for 3-year patient outcomes (Figure 4(g)).

### 3.5. External Validation of the Gene Prognostic Model

**Figure 5:**
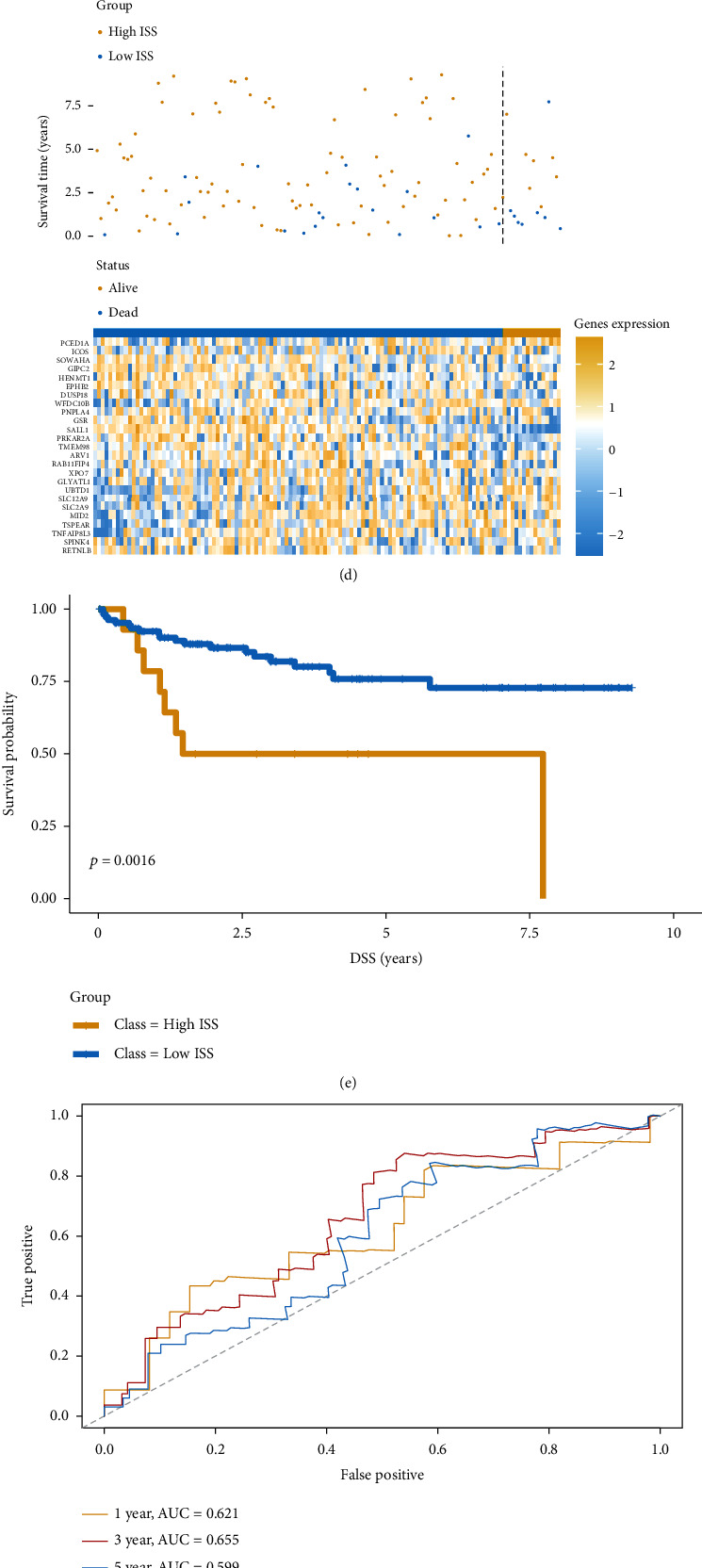
ISS as a predictor of patient prognosis in the validation cohorts: (a) distribution of high- and low-ISS groups in the GSE17536 cohort; (b) comparison of patient prognosis between the high- and low-ISS groups of the GSE17536 cohort; (c) ROC curve for ISS in the GSE17536 cohort; (d) distribution of the high- and low-ISS groups in the GSE38832 cohort; (e) comparison of patient prognosis in the high- and low-ISS groups of the GSE38832 cohort; (f) ROC curve for ISS in the GSE38832 cohort; (g) distribution of the high- and low-ISS groups in the GSE39582 cohort; (h) comparison of patient prognosis between the high- and low-ISS groups of the GSE39582 cohort; (i) ROC curve for ISS in the GSE39582 cohort.

Three external datasets, namely, GSE17536, GSE38832, and GSE39582, were used to validate the gene prognostic model. Patients were divided into high- and low-ISS groups to verify the efficiency of the model (Figures 5(a), 5(d), and 5(g)). Kaplan- Meier survival curves of the three datasets revealed significant differences in OS between the groups. High-ISS groups had markedly shorter duration of OS than the low-ISS groups (Figures 5(b), 5(e), and 5(h)). On ROC analysis in GSE17536, the areas under the curve (AUCs) for 1-, 3-, and 5-year OS were 0.618, 0.587, and 0.591, respectively (Figure 5(c)). In GSE38832, the AUCs for 1-, 3-, and 5- year OS were 0.621, 0.655, and 0.599, respectively (Figure 5(f)). In GSE39582, the corresponding AUCs were 0.552, 0.603, and 0.582, respectively (Figure 5(i)). These results confirmed the validity of our model. External validation indicated that the model performed well in predicting outcomes in CRC patients.

### 3.6. ISS Had Considerable Potential in Predicting the Immune Features and Immunotherapy Responsiveness of CRC

**Figure 6:**
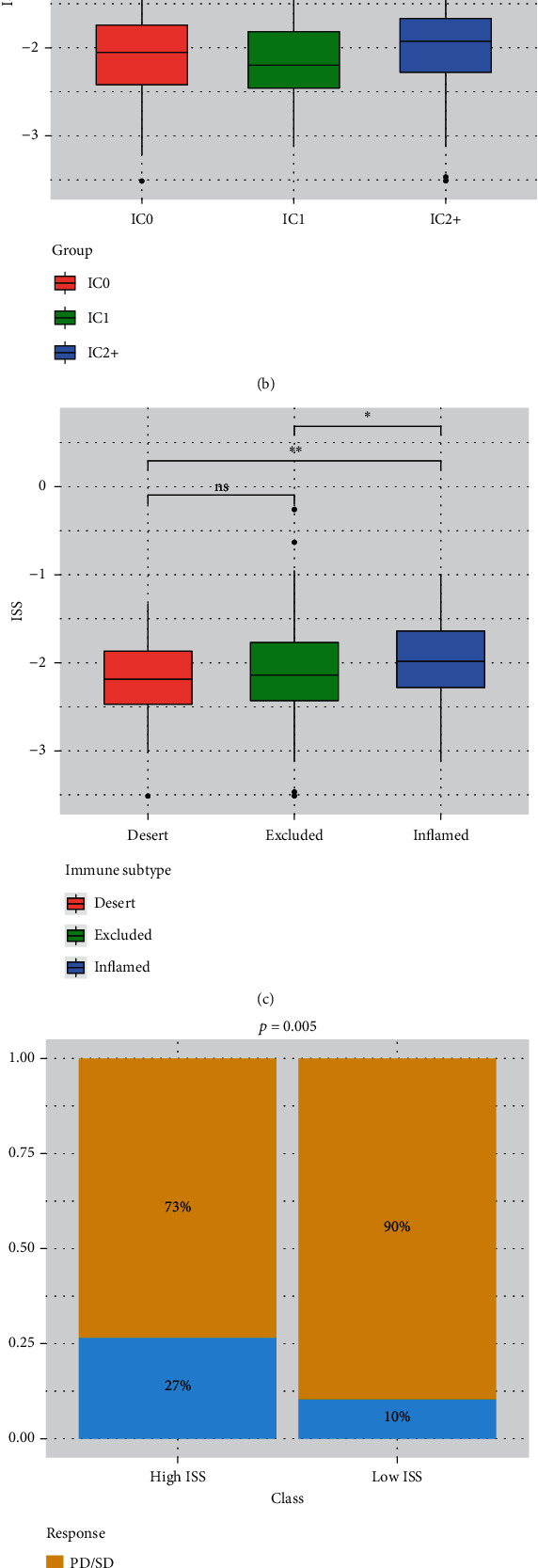
ISS as a predictor of tumor immune status and immunotherapy responsiveness: (a) correlation between the ISS and ISS component genes and tumor mutation burden and immune features; (b) comparison of ISS between different immune cell groups; (c) comparison of ISS between different immune subtypes; (d) comparison of immunotherapy responsiveness between the high- and low-ISS groups; (e) comparison of patient prognosis between the high- and low-ISS groups.

We subsequently analyzed the correlation between cancer stemness, immune checkpoint expression, immune infiltration levels, and ISS and ISS component genes (Figure 6(a)). We found that the expression level of the MID2 gene correlated positively with that of all immune checkpoints and cancer stemness (Figure 6(a)). In addition, the ISS predicted the immune cell subgroup and immune microenvironment subtype in the immunotherapy cohort (Figures 6(b) and 6(c)). Our results also demonstrated that high ISS was predictive of better immunotherapy responsiveness and outcomes during immunotherapy (Figures 6(d) and 6(e)).

## 4. Discussion

CRC remains a global health problem. As conventional markers and/or staging methods do not appropriately address the heterogeneity of CRC, novel prognostic models are urgently needed for improving patient outcomes.

Somatic mutations play an important role in CRC [26, 27]. The ten most common genes with high mutation frequency in CRC were screened in this study; these included APC, TP53, TTN, KRAS, SYNE1, MUC16, PIK3CA, FAT4, RYR2, and OBSCN. We then evaluated the association between clinical outcomes and these genes. Our findings suggested that only TTN mutations were found to be negatively associated with outcomes in CRC patients; this indicated that TTN may play an important role in CRC (Figure 1). TTN transcribes a mRNA that encodes titin. TTN mutations have been found to be associated with various diseases including dilated cardiomyopathy, centronuclear myopathy, and squamous cell carcinoma of the lung, among others [28–30].

We identified DEGs between TTN-mutated and wild-type CRC and compared these groups; 88 and 115 genes in the TTN-mutant group were upregulated and downregulated, respectively. Enrichment analysis was performed using these genes. The upregulated genes were enriched in various pathways including the viral protein interaction with cytokine and cytokine receptor, T helper 17 cell differentiation, and tumor necrosis factor signaling pathways, among others. The downregulated genes were enriched in the Wnt signaling and PPAR signaling pathways, among others. As some of the enriched pathways were related to the immune system [18, 31, 32], we evaluated the expression of immune checkpoints in the TTN-mutant and wild-type groups. The expression of CD274, CTLA4, HAVCR2, LAG3, PDCD1, PDCD1LG2, and TIGIT was higher in the TTN-mutant group than in the wild-type group. In addition, high CD8+ infiltration levels were observed in the TTN-mutant group. This finding suggested that TTN mutation-mediated tumor microenvironments are associated with immune inflamed tumor subtypes and may show high responsiveness to immunotherapy.

We further analyzed the immune status of the identified 3 subtypes. The immune suppressive subtype demonstrated the highest levels of CD274, CTLA4, HAVCR2, LAG3, PDCD1, PDCD1LG2, and TIGIT expression. In addition, the immune suppressive subtype had a higher level of CD8+ T cell and immune suppressive cell (such as Treg cells) infiltration compared to the immune active subtype. In this tumor microenvironment, CD8+ T cell will perform the dysfunction phenotype under the microenvironment immune-suppressive factors (immune checkpoints, immune suppressive cell). This phenotype has the high potential to be reversed by the immune checkpoint blockades. These results indicated that the immune suppressive subtype may potentially be sensitive to immunotherapy.

We subsequently acquired the DEGs between the immune suppressive and immune active subtypes and constructed an immune feature-based prognostic model to predict the outcomes and immune status of these patients. Some of the ISS component genes showed prognostic potential in CRC. For example, elevated Rab11-family interacting protein 4 (encoded by RAB11FIP4) expression in CRC tissues was associated with poor prognosis [33]. A reduction in SPINK4 expression in CRC tissues was also associated with poor prognosis [34]. We further validated the prognostic efficiency of ISS in external datasets to test model robustness; the findings showed that our prognostic model successfully predicted the prognosis of patients in both training and validation sets.

In this context, the characteristics of the immune microenvironment decide responsiveness to immunotherapy [13, 35–37]. During analysis, we found that the ISS and its component genes demonstrated correlation with features including cancer stemness, immune infiltration, and immune checkpoint expression. In the immunotherapy cohort, ISS could effectively predict patient outcomes and responsiveness to immunotherapy. These results indicated that the ISS has considerable potential for clinical application to identify candidates suitable for immunotherapy.

Meanwhile, MID2 was the ISS component gene with the second highest coefficient and significantly positively correlated with the immune checkpoint expression. Recent research has demonstrated that MID2 plays an essential role in breast and ovarian cancer progression and can be a potential cancer biomarker [38–40]. Our findings suggest that MID2 has the great potential to be explored as the maker of immune checkpoint blockade response and cancer stemness.

## 5. Conclusion

In this study, we constructed a TTN mutation-related and immune feature-based novel prognostic model for CRC patients. This prognostic model was related to the microenvironment status of CRC and may be used as an indicator for individualized therapy; the model may also be used in predicting the clinic benefit obtained from immunotherapy.

## Data Availability

Data are available in public, open-access repositories. Detailed information can be acquired in contacting the corresponding author.

## Supplementary Materials

Supplementary 1 Figure S1: enrichment analysis for DEGs between TTN-mutant and wild-type tumors. a: enrichment analysis for upregulated genes based on the KEGG database. b: enrichment analysis for upregulated genes based on the GO database. c: enrichment analysis for downregulated genes based on the KEGG database. d: enrichment analysis for downregulated genes based on the GO database.

Supplementary 2 Figure S2: enrichment analysis for DEGs between immune suppressive and immune active CRC subtypes. a: enrichment analysis for upregulated genes based on the KEGG database. b: enrichment analysis for upregulated genes based on the GO database. c: enrichment analysis for downregulated genes based on the KEGG database. d: enrichment analysis for downregulated genes based on the GO database.
