# Supplementary figures and images for: Construction of a TTN Mutation-Based Prognostic Model for Evaluating Immune Microenvironment, Cancer Stemness, and Outcomes of Colorectal Cancer Patients
Source: Stem Cells Int. 2023 Feb 21;2023:6079957. doi: 10.1155/2023/6079957 (PMC9990748; doi:10.1155/2023/6079957)

A

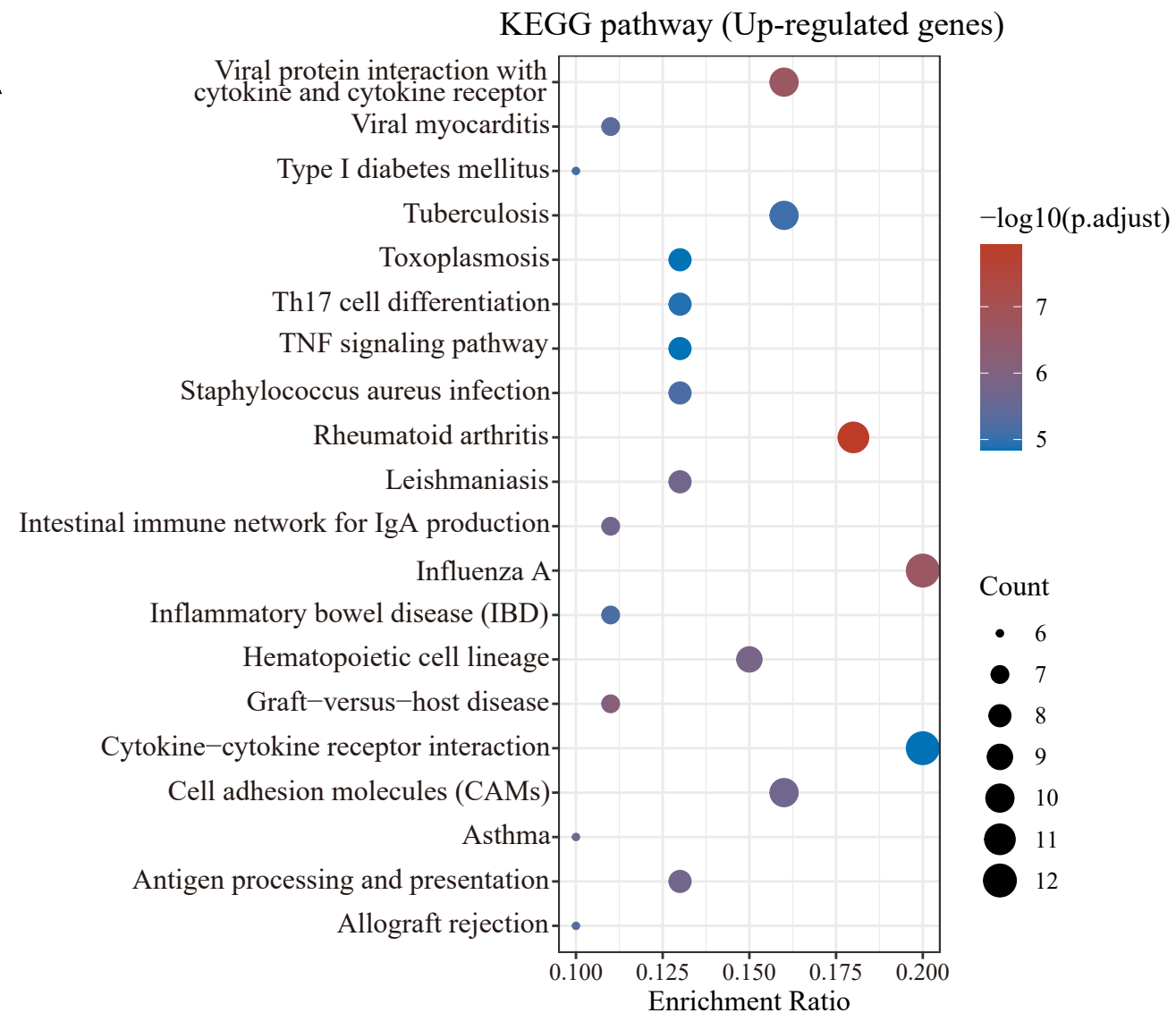

B

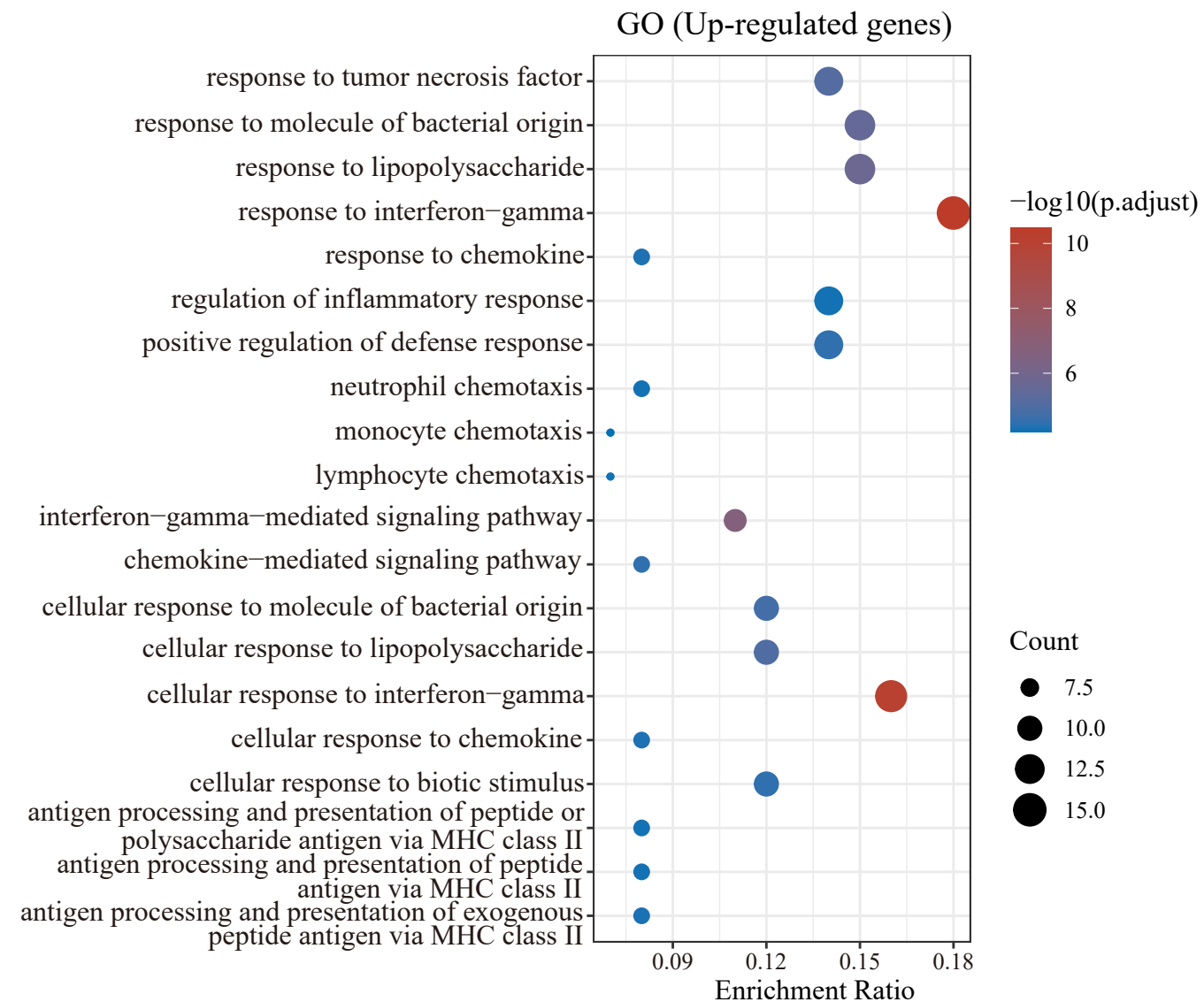

C

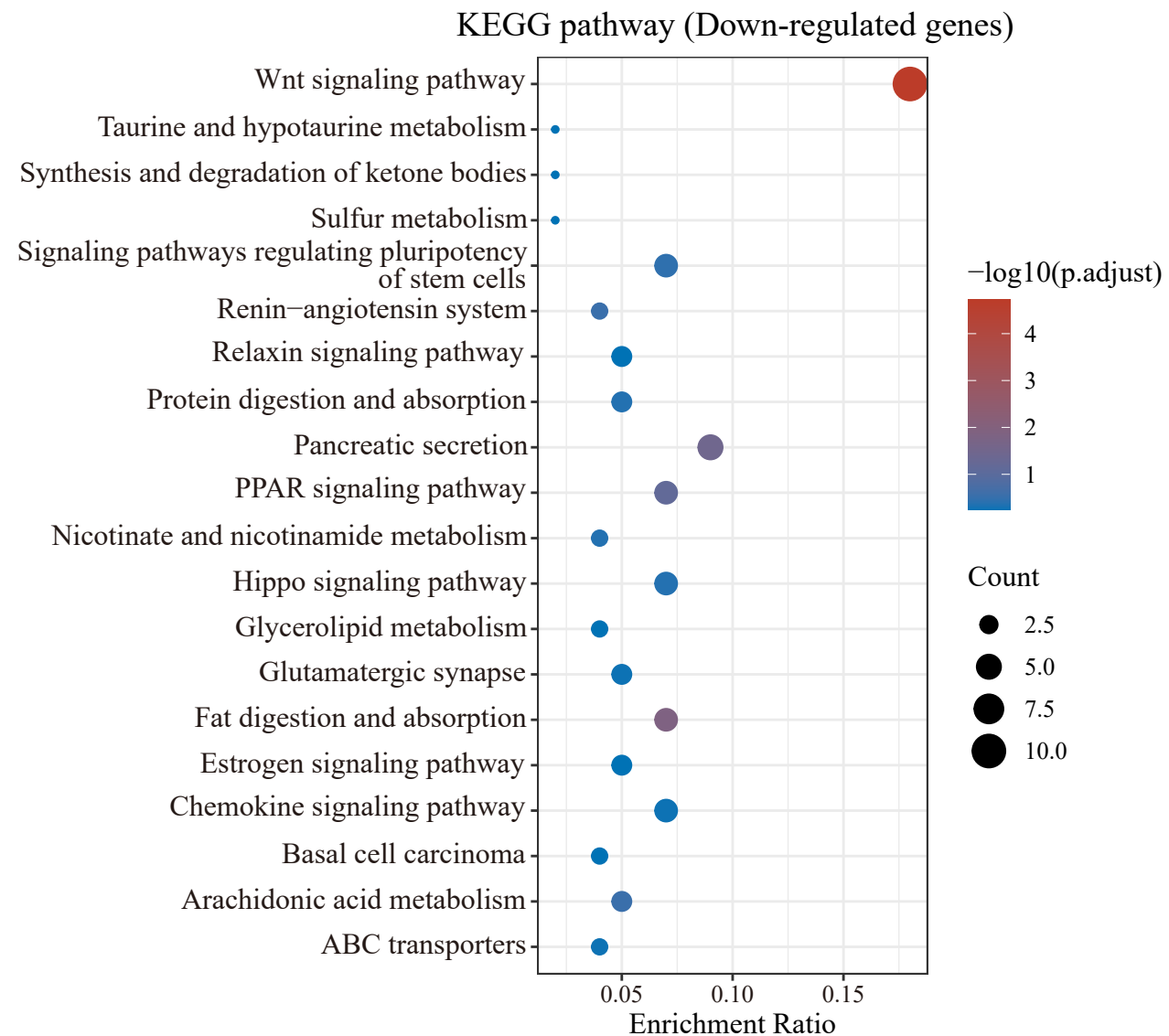

D

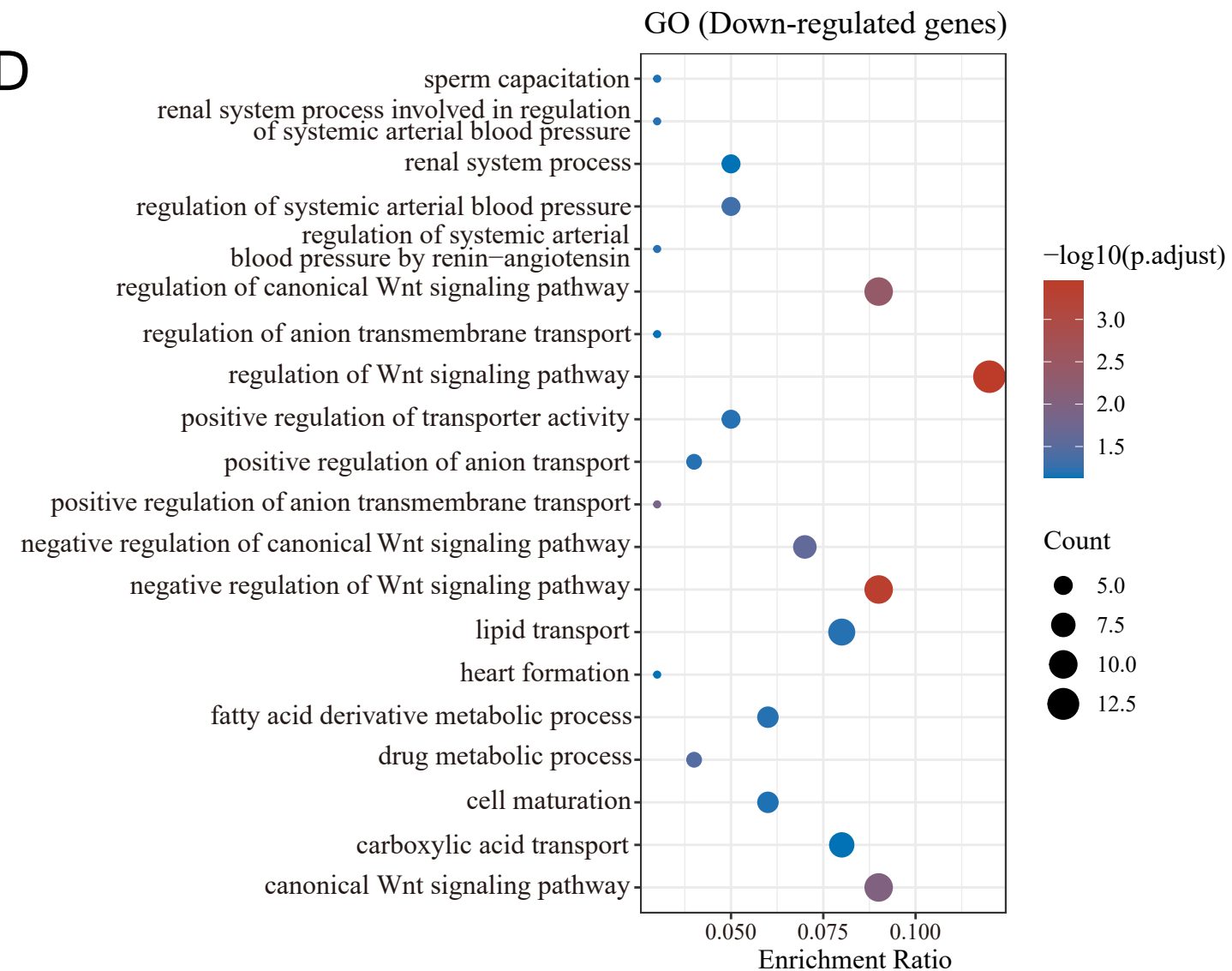

Supplement: Supplementary 1 — Figure S1: enrichment analysis for DEGs between TTN-mutant and wild-type tumors. a: enrichment analysis for upregulated genes based on the KEGG database. b: enrichment analysis for upregulated genes based on the GO database. c: enrichment analysis for downregulated genes based on the KEGG database. d: enrichment analysis for downregulated genes based on the GO database. [file 6079957.f1.pdf]
