# Supplementary material for: Construction of a TTN Mutation-Based Prognostic Model for Evaluating Immune Microenvironment, Cancer Stemness, and Outcomes of Colorectal Cancer Patients
Source: Stem Cells Int. 2023 Feb 21;2023:6079957. doi: 10.1155/2023/6079957 (PMC9990748; doi:10.1155/2023/6079957)
Supplement: Supplementary 2 — Figure S2: enrichment analysis for DEGs between immune suppressive and immune active CRC subtypes. a: enrichment analysis for upregulated genes based on the KEGG database. b: enrichment analysis for upregulated genes based on the GO database. c: enrichment analysis for downregulated genes based on the KEGG database. d: enrichment analysis for downregulated genes based on the GO database. [file 6079957.f2.pdf]

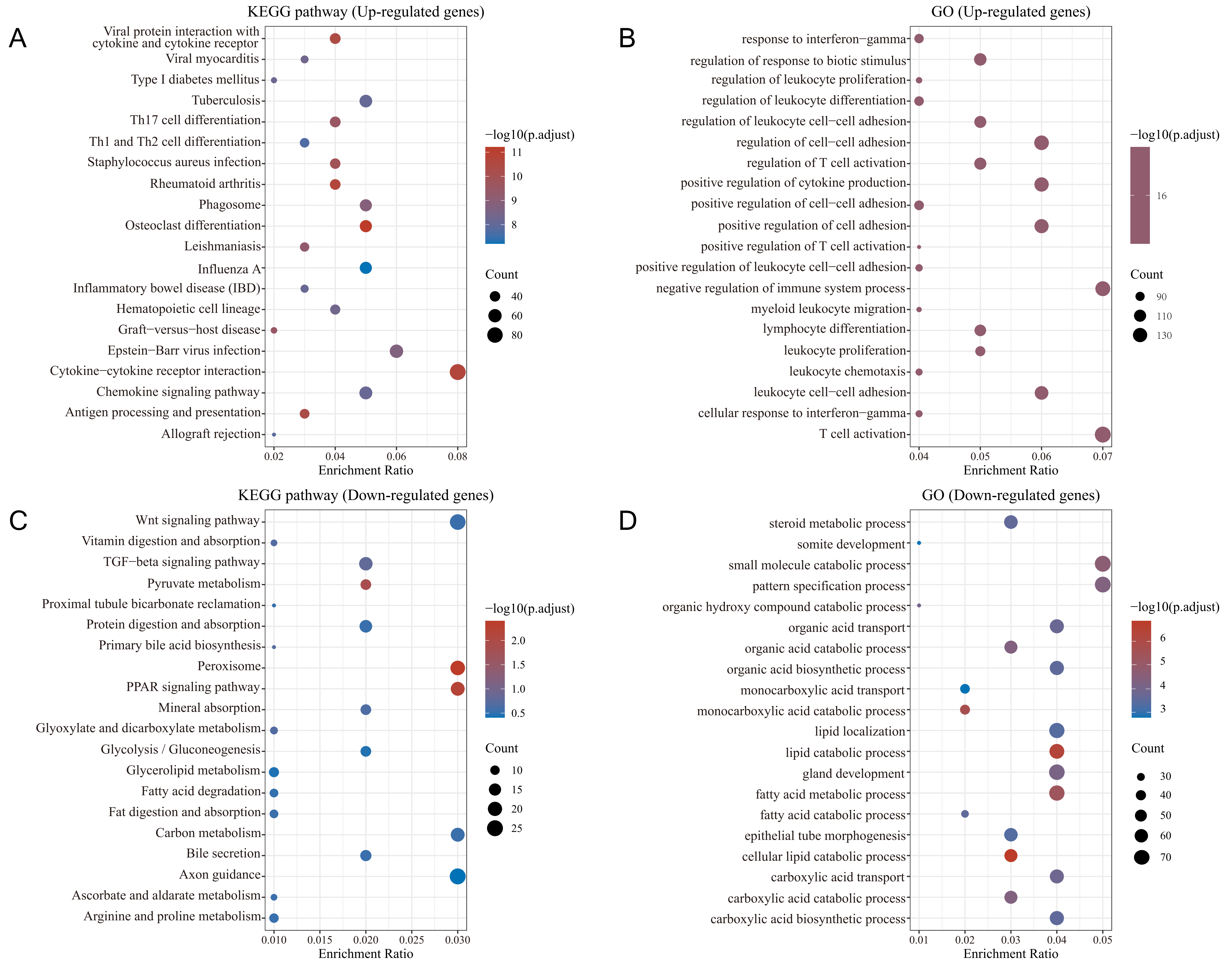
A
KEGG pathway (Up-regulated genes)
Viral protein interaction with cytokine and cytokine receptor
Viral myocarditis
Type I diabetes mellitus
Tuberculosis
Th17 cell differentiation
Th1 and Th2 cell differentiation
Staphylococcus aureus infection
Rheumatoid arthritis
Phagosome
Osteoclast differentiation
Leishmaniasis
Influenza A
Inflammatory bowel disease (IBD)
Hematopoietic cell lineage
Graft−versus−host disease
Epstein−Barr virus infection
Cytokine−cytokine receptor interaction
Chemokine signaling pathway
Antigen processing and presentation
Allograft rejection
0.02
0.04
0.06
0.08
Enrichment Ratio
−log10(p.adjust)
11
10
9
8
Count
40
60
80
B
GO (Up-regulated genes)
response to interferon−gamma
regulation of response to biotic stimulus
regulation of leukocyte proliferation
regulation of leukocyte differentiation
regulation of leukocyte cell−cell adhesion
regulation of cell−cell adhesion
regulation of T cell activation
positive regulation of cytokine production
positive regulation of cell−cell adhesion
positive regulation of cell adhesion
positive regulation of T cell activation
positive regulation of leukocyte cell−cell adhesion
negative regulation of immune system process
myeloid leukocyte migration
lymphocyte differentiation
leukocyte proliferation
leukocyte chemotaxis
leukocyte cell−cell adhesion
cellular response to interferon−gamma
T cell activation
0.04
0.05
0.06
0.07
Enrichment Ratio
−log10(p.adjust)
16
Count
90
110
130
C
KEGG pathway (Down-regulated genes)
Wnt signaling pathway
Vitamin digestion and absorption
TGF−beta signaling pathway
Pyruvate metabolism
Proximal tubule bicarbonate reclamation
Protein digestion and absorption
Primary bile acid biosynthesis
Peroxisome
PPAR signaling pathway
Mineral absorption
Glyoxylate and dicarboxylate metabolism
Glycolysis / Gluconeogenesis
Glycerolipid metabolism
Fatty acid degradation
Fat digestion and absorption
Carbon metabolism
Bile secretion
Axon guidance
Ascorbate and aldarate metabolism
Arginine and proline metabolism
0.010
0.015
0.020
0.025
0.030
Enrichment Ratio
−log10(p.adjust)
2.0
1.5
1.0
0.5
Count
10
15
20
25
D
GO (Down-regulated genes)
steroid metabolic process
somite development
small molecule catabolic process
pattern specification process
organic hydroxy compound catabolic process
organic acid transport
organic acid catabolic process
organic acid biosynthetic process
monocarboxylic acid transport
monocarboxylic acid catabolic process
lipid localization
lipid catabolic process
gland development
fatty acid metabolic process
fatty acid catabolic process
epithelial tube morphogenesis
cellular lipid catabolic process
carboxylic acid transport
carboxylic acid catabolic process
carboxylic acid biosynthetic process
0.01
0.02
0.03
0.04
0.05
Enrichment Ratio
−log10(p.adjust)
6
5
4
3
Count
30
40
50
60
70
